# Assessing ultrasonographic optic nerve sheath diameter in animal model with anesthesia regimens

**DOI:** 10.1590/acb370308

**Published:** 2022-06-15

**Authors:** Maira de Robertis Azevedo, Marcelo de-Lima-Oliveira, Alessandro Rodrigo Belon, Sérgio Brasil, Manoel Jacobsen Teixeira, Wellingson Silva Paiva, Edson Bor-Seng-Shu

**Affiliations:** 1 MSc. Universidade de São Paulo – School of Medicine – Hospital das Clínicas – Division of Neurological Surgery – São Paulo (SP), Brazil.; 2 MD. PhD. Universidade de São Paulo – School of Medicine – Hospital das Clínicas – Division of Neurological Surgery – São Paulo (SP), Brazil.; 3 PhD. Universidade de São Paulo – School of Medicine – Hospital das Clínicas – Laboratory for Experimental Surgery – SãoPaulo (SP), Brazil.

**Keywords:** Intracranial Pressure, Intracranial Hypertension, Experimental Research

## Abstract

**Purpose:**

To determine the normal optical nerve sheath (ONS) diameter ultrasonography (ONSUS) and evaluate the possible effects of drugs on ONS diameter during anesthetic induction in healthy pigs.

**Methods:**

Healthy piglets were divided into three groups: a control group, that received xylazine and ketamine (X/K); other that received xylazine, ketamine and propofol (X/K/P); and a third group that received xylazine, ketamine, and thiopental (X/K/T). The sheath diameter was assessed by ultrasonography calculating the average of three measurements of each eye from the left and right sides.

**Results:**

118 animals were anesthetized (49 X/K 33 X/K/P and 39 X/K/T). Mean ONS sizes on both sides in each group were 0.394 ± 0.048 (X/K), 0.407 ± 0.029 (X/K/P) and 0.378 ± 0.042 cm (X/K/T) (medians of 0.400, 0.405 and 0.389, respectively). The ONS diameter varied from 0.287–0.512 cm (mean of 0.302 ± 0.039 cm). For group X/K, the mean diameter was 0.394 ± 0.048 cm. Significant differences in ONS sizes between the groups P and T (X/K/P > X/K/T, p = 0.003) were found. No statistically significant differences were detected when other groups were compared (X/K = X/K/P, p = 0.302; X/K = X/K/T, p = 0.294).

**Conclusions:**

Sedation with thiopental lead to significative ONS diameter reduction in comparison with propofol. ONSUS may be useful to evaluate responses to thiopental administration.

## Introduction

Intracranial pressure (ICP) is usually monitored using catheters located in different areas of the brain. These devices are invasive and can cause complications, including infections and bleeding. However, estimation and monitoring of ICP is feasible by means of noninvasive techniques^1^^-^ 4.

The optical nerve is part of the central nervous system and is surrounded by a sheath containing cerebrospinal fluid derived from the subarachnoid space. Because the subarachnoid space is contiguous with the encephalon, changes in the ICP can affect the optical nerve sheath (ONS) and cause variations in its diameter^5^. Optical nerve sheath diameter ultrasonography (ONSUS) is a reliable method for the noninvasive estimation of ICP because it is highly sensitive to variations in sheath diameter^6^. Current experimental studies have demonstrated the increase of ONS during intracranial hypertension^7^^,^ 8. However, there is no standardized optical sheath nerve measurement in piglets, which have some physiological similarities with humans.

The current study sought to determine the normality of the diameter of the ONSUS among healthy piglets without intracranial hypertension using ultrasound and to evaluate the possible effects of drugs on the sheath diameter during anesthetic induction.

## Methods

The Animal Research Ethics Committee of the School of Medicine at the Universidade de São Paulo approved this study under Protocol No. 019/14.

Healthy hybrid pigs from the Landrace, Pietrain, and Duroc breeds weighing ± 2.5 kg were evaluated for 3 months. The experiment was taken on the Laboratory for Experimental Surgery of USP.

### Study animals

The pigs underwent 12 h of fasting with free access to water until 1 h before the experiment.

The animals were carefully handled in transport cages to avoid injury, and the following preanesthetic medications were administered intramuscularly shortly after the cages were opened: ketamine (Ketamin-S Cristália) at a dose of 10 mg/kg and xylazine (Anasedan) at a dose of 2 mg/kg. After the animals presented with normal stress levels, they were placed in the lateral-lateral position on a surgical table to begin an ultrasound optical sheath nerve examination without general anesthesia.

After a 15-min examination, the marginal vein of the left ear was catheterized using a 20- or 22-gauge vascular catheter (BD Insyte). After venous access was achieved, the animals received a saline solution (0.9% NaCl) at 20 mg/kg to compensate for the volume loss during fasting. After this procedure, general anesthesia was performed. ONSUS was performed in two opportunities with an average of 10 minutes of interval. The first measure at sedation induction with xylazine and ketamine, the second immediately after endotracheal intubation and administration of propofol or thiopental in concentrations adjusted for animals’ weight in order to produce deep sedation^4^.

The animals were divided into three anesthetic induction groups: control group that received xylazine and ketamine (X+K); other group that received xylazine, ketamine, and propofol (X+K+P), and a third group that received with xylazine, ketamine, and thiopental (X+K+T). Propofol (Provine 1%, Cláris) was administered at a dose of 5 mg/kg, and thiopental (Thiopentax) was administered at a dose of 12.5 mg/kg. The animals were intubated using an endotracheal probe of diameter 6 (Portex).

The central temperature of the animals was maintained at 37–38 °C (the normal temperature for pigs) using a thermal mattress and previously heated maintenance solutions.

### Optic nerve measurements

An ultrasound device (SonoSite-Micromax, FUJIFILM, Washington DC, USA) was coupled with a high-frequency linear transducer (6–13 MHz and SLAx SonoSite model), which was carefully placed on the upper eyelid of the pigs.

**Figure 1:**
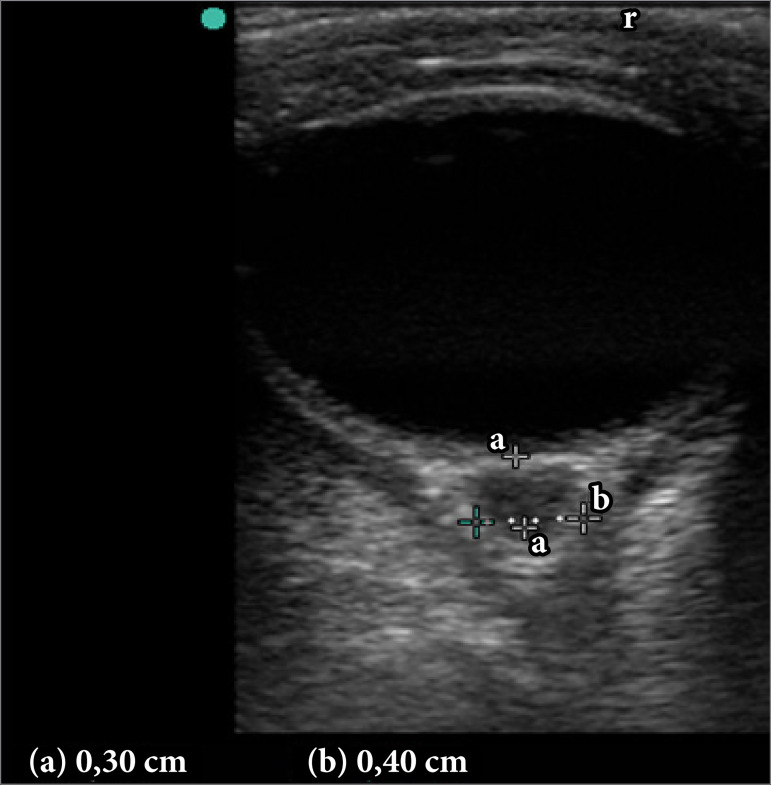
Technique for eye insonation. A 3-mm straight line from the optic disc **(a)** is drawn, and the optic nerve sheath diameter is measured between the hyperechoic columns **(b)**.

Images of the eyeball were acquired in the horizontal or vertical planes depending on its rotation. The measures were as follows: (a) the distance between the center of the optical disc found by pulling the cursor 0.30 cm down toward the optic nerve and (b) the distance between the walls of the nerve sheath (Fig. 1). Imaging model was the same used in a previous study^5^. The sheath diameter was measured by calculating the average of three measurements of each eye, beginning on the left side and ending on the right side, according to the study protocol.

### Statistical analyses

Descriptive statistical analyses were initially conducted by calculating the mean, median, minimum, and maximum values; standard deviation, absolute, and relative frequencies (percentages); and one-dimensional scatterplots and individual profiles (lines).

The inferential analyses used to confirm or reject the evidence found in the descriptive analysis included the following: Pearson’s chi-square test for comparing the sex distribution among the study groups (X+K, X+K+P, and X+K+T); the Kruskal–Wallis test for comparing the weight (kg) between the anesthesia study groups (X+K, X+K+P, and X+K+T). A fixed-factor analysis of variance (ANOVA)^9^ for comparing the optic nerve diameter (cm) among the study groups (X+K, X+K+P, and X+K+T) and multiple-comparison tests using Tukey’s test and Dunnett’s test where necessary. The level of significance was set to 5% for all analyses. All data entered into Microsoft Excel for later analysis. All statistical analyses were conducted using R version 3.0.2.

## Results

A total of 118 healthy hybrid piglets (54 males and 64 females) from the Landrace, Pietrain, and Duroc breeds, aged 40 to 70 days and weighing approximately 20 kg each, were anesthetized. Forty-nine animals were anesthetized with X+K, 33 animals were anesthetized with X+K+P, and 36 animals were anesthetized with X+K+T. Table 1 shows the analysis of each group by weight, sex, and number of animals.

**Table 1:** Gender and weight (kg) distribution of the animals in each group.

Distribution		Anesthetic Group									Total			P
		X+K			X+K+P			X+K+T						
Gender		n			n			n			n			
	Female	30		61.2%	17		51.5%	17		47.2%	64		54.2%	0.411^a^
	Male	19		38.8%	16		48.5%	19		52.8%	54		45.8%	
	Total	49		100.0%	33		100.0%	36		100.0%	118		100.0%	
Weight (kg)														
	N		49			33			36			118		0.068^b^
	Mean		18.3			18.4			17.5			18.1		
	Median		18.0			18.6			17.1			18.0		
	S.D.		±2.8			±1.9			±2.8			±2.6		

a Pearson’s chi-squared,

b Kruskal–Wallis, S.D: standard deviation.

The diameter of the ONS on both sides were 0.394 ± 0.048 cm (0.400 cm) for the X+K group; 0.407 ± 0.029 cm (0.405 cm) for the X+K+P group, and 0.378 ± 0.042 cm (0.389) for the X+K+T group (Table 2). Considering all groups, the diameter of the ONS varied from 0.287–0.512 cm (mean of 0.302 ± 0.039 cm).

**Table 2:** Measurements of optic nerve diameter (cm) of the animals of each group.

Anesthetic group		Left side	Right side	Mean of sides
X+K	n	49	49	49
	mean	0.393	0.394	0.394
	SD	0.050	0.049	0.048
**X+K+P**	**n**	**33**	**33**	**33**
	mean	0.403	0.412	0.407
	SD	0.030	0.032	0.029
**X+K+T**	**n**	**36**	**36**	**36**
	mean	0.377	0.378	0.378
	SD	0.047	0.040	0.042
	p	0.047^c^	0.005^c^	0.011^c^
				

c Analysis of variance (ANOVA) with a fixed factor.

The comparison among these three groups indicated that the optic nerve sheath diameter (ONSD) was smaller in the X+K+T group than the X+K+P group (p = 0.003). However, no significant difference in diameter was found between the X+K and X+K+P groups (p = 0.302) or between the X+K and X+K+T groups (p = 0.294). The results indicated a significant difference between the groups X+K+T and X+K+P; the mean diameter for the group anesthetized with thiopental was significantly lower than that for the group anesthetized with propofol (Table 3).

**Table 3:** Results of multiple comparisons between groups anesthetics.

Left side			Right side			Mean	
Conclusion	p		Conclusion	p		Conclusion	P
X+K = X+K+P	0.661^d^		X+K = X+K+P	0.138^e^		X+K = X+K+P	0.302^d^
							
X+K = X+K+T	0.343^d^		X+K = X+K+T	0.212^e^		X+K = X+K+T	0.294^d^
							
X+K+P > X+K+T	0.028^d^		X+K+P > X+K+T	0.003^e^		X+K+P > X+K+T	0.003^d^

d Dunnett method

e Tukey method.

## Discussion

Ultrasonography of the ONSD can be used to noninvasively assess ICP changes, and it is frequently used in situations in which invasive methods are unavailable or contraindicated^10^^,^ 11. This method is simple and noninvasive and can be repeated multiple times for reassessment without causing harm to the patient. Diameter changes usually detect ICP elevations with satisfactory sensitivity in cases in which the sheath diameters are above the normal reference values^10^^,^ 12 ^,^ 13.

No studies to date have used pigs as a model to standardize the normal values for the ONSUS and compare with models of intracranial hypertension. This study with 118 animals included 708 measurements, and the ONSD varied from 0.287 cm to 0.512 cm, considering all groups (mean of 0.302 ± 0.039 cm). For group X+K (which did not receive drugs that could affect ICP), the diameter was 0.394±0.048 cm (Table 2). To the best of our knowledge, there are no data about normal piglet ONS in the literature. Some authors have demonstrated the ability of ONSUS to detect acute ICP increases^12^^,^ 14. However, they compared the basal measurement in a small group of adult pigs without a standard measurement^15^.

The present study may provide a size range for the ONS in piglets, which are currently the main models for studying many subjects, including ICP.

**Figure 2:**
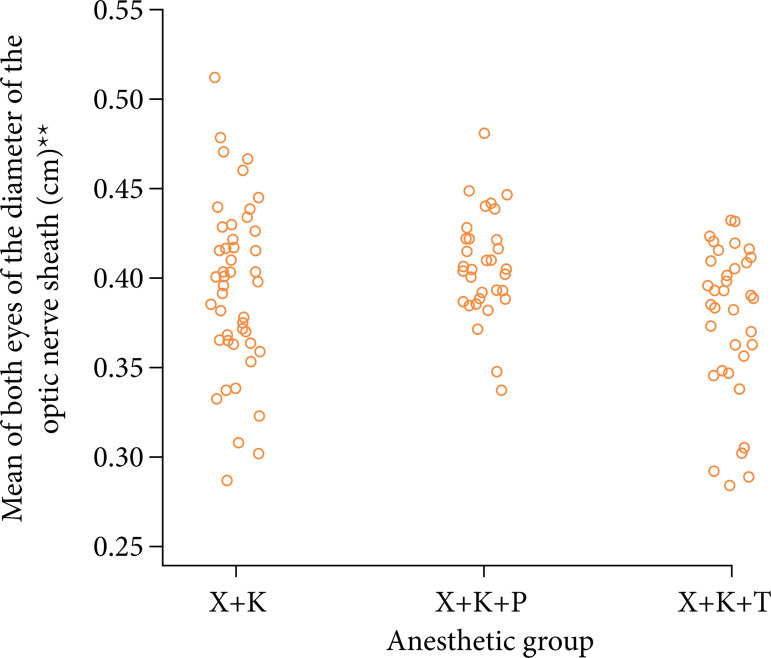
One-dimensional dispersion diagram of the mean optic nerve diameter (cm) of the animals. **mean of six measures (three on each side).

The smaller diameter in the former group might be associated with the ability of thiopental to decrease ICP by reducing blood flow, cerebral metabolism, or both. In this respect, whether the reduction in cerebral blood flow due to thiopental is a consequence of the reduced metabolic demand of the neuronal tissue is unclear^16^. However, a previous study suggested that thiopental is a potent cerebral vasoconstrictor that decreases ICP by reducing cerebral blood flow^17^. Thiopental has a more pronounced effect on cerebral microvascular vasoconstriction than propofol^18^^,^ 19. Therefore, the results of this study suggest that the reduction in ICP is higher among animals anesthetized with thiopental, although ICP was assumed normal before light anesthesia with pre-anesthesia. However, no significant difference was found between the groups X+K and X+K+T, although the scatterplot showed that the diameter tended to be higher in the X+K group (Fig. 2).

Moreover, the mean diameter of the sheath in the X+K group was not significantly higher than that in the X+K+T group (0.394 ± 0.048 cm vs. 0.378 ± 0.042 cm). The comparison of measurements after anesthetic induction (i.e., after using a higher dose of thiopental for anesthetic maintenance during deep anesthesia) might yield significant differences between these groups. However, the data were collected only during anesthetic induction.

Importantly, this study was the first to standardize the measurements of the optic nerve of pigs, which are the experimental models often used to analyze intracranial hypertension. Therefore, future studies on the ONSUS using these experimental models might provide normal reference values. In addition, these results indicated a reduction in sheath diameter in sedated animals using anesthetic drugs known to reduce ICP, even in those with supposedly normal ICP. This latter finding might confirm the sensitivity of the measurement of the ONS in detecting ICP changes, even in the absence of intracranial hypertension.

### Limitations

Since it is not possible to measure ONSD in awake animals for obvious reasons, all three groups received ONSUS evaluations under the effects of xylazine and ketamine at the baseline, what may not indicate exactly the true ONS diameter cut-offs at rest but provide values for studies with animals under general anesthesia. The observation of long duration infusion of these sedatives over the ONS diameter was not analyzed, because these animals undergone ICP manipulation posterior to these assessments, being its results published elsewhere^5^.

Another limitation refers to the possibility of ketamine leading to uncontrollable ICP. The postulated mechanism surrounds large vessel vasodilation from an elevation in pCO_2_ in nonventilated patients, and the small vessel vasoconstriction effects related to ketamine’s nitric oxide synthase inhibition leading to a potential increase in cerebral oxygen extraction^20^^,^ 21. However, this particular behavior was observed in patients where there was concern for elevated ICP, differently of this study’s population. Finally, ONSUS was performed by a single operator and there is lack of interobserver comparison.

## Conclusion

Sedation with thiopental lead to significative ONS diameter reduction in comparison with propofol. ONSUS seems to be suitable to evaluate responses to thiopental administration.
